# Network coding construction for a special class of three unicast sessions

**DOI:** 10.1038/s41598-023-47203-8

**Published:** 2023-11-20

**Authors:** Baoxing PU

**Affiliations:** School of Data Science and Software Engineering, Wuzhou University, Wuzhou, 543002 China

**Keywords:** Electrical and electronic engineering, Computer science

## Abstract

For a special class of three unicast sessions, in which the maximum flow from each sender to each receiver is the same positive integer *k*, a network coding approach is proposed. A multigeneration mixed strategy, in which (2 × *n* + 1) consecutive generations are taken as a mixed set, is adopted. The precoding strategy is adopted at the senders, random linear network coding technology is adopted at intermediate nodes to transmit data, and interference alignment technology is used at the receivers to eliminate some interference information. The proposed approach can construct a network coding data transmission scheme, in which the transmission rate vector of the senders is [(*n* + 1) × *k*/(2 × *n* + 1), *n* × *k*/(2 × *n* + 1), *n* × *k*/(2 × *n* + 1)]. The feasibility of the proposed approach is mathematically deduced and proven, and the simulation results verify the conclusions of the theoretical analysis.

## Introduction

Network coding^1^ is a new data transmission technology that was initially studied for single sender multicast networks and has achieved good research results in this field^2–5^.

Compared to routing transmission technology, network coding allows network nodes to encode the received data before transmitting it to the output channel. Network coding has advantages in improving network throughput, reducing network energy consumption, improving network robustness and security^6^. Since its inception, this technology has been extensively studied and has been proven to be applicable in multiple fields, such as single source multicast, multisource multicast, wireless networks, large capacity file distribution, cloud storage, and the Internet of Things^7–11^. Reference^12^ points out that network coding is a key technology to meet the growing demand of future networks.

For widely used linear network coding, the output channel of a node transmits the encoding of the node's input data. This encoding is a linear combination of input data, and the coefficients of this linear combination are called local encoding vector. Therefore, each output channel corresponds to a local encoding vector. The local encoding vectors of all output channels of all nodes form a network coding data transmission scheme. Before using network encoding for data transmission, it is necessary to design a local encoding vector for each output channel of each node, which is called constructing a network coding data transmission scheme^4,5^.

In practical applications, most communication networks appear in the form of multiple senders. Driven by application requirements, scholars have gradually turned to the study of multisender network coding^8^. However, the construction of multisender network coding is a very difficult problem. To date, although some research results have been obtained, no substantive breakthroughs have been made. Among them, there is a special multisender network coding problem named multiunicast network coding^7^, which is relatively simple and accounts for a large proportion of practical applications. The research in Ref.^13^ showed that any directed acyclic network (including a multisender network) can construct a corresponding multiunicast network, and the original network and the multiunicast network have the same network coding solvability. In other words, if there is a feasible linear network coding data transmission scheme in the constructed multiunicast network, there must be a feasible linear network coding data transmission scheme in the original network. Therefore, multiunicast network coding research, on the one hand, is necessary for network coding technology applied to multiunicast networks; on the other hand, it also provides an effective way to solve the general multisender network coding problem. As a result, research on multiunicast network coding has attracted the attention of the academic community.

In Ref.^14^, it was noted that it is also quite difficult to solve the multiunicast network coding problem. Even the simplest dual unicast network coding is an NP problem. In practical applications, if network coding technology is used to realize data transmission in a multiunicast network, then constructing a network coding data transmission scheme is an essential step. Therefore, the construction of multiunicast network coding is a research hotspot.

In view of the difficulty of multiunicast network coding, most of the existing research work only involves the case of few senders (dual unicast network and three unicast networks). Through research on special and simple problems, researchers have attempted to solve general problems^15–17^. For existing research on three-unicast network coding, some researchers adopted heuristic algorithms, and others only focused on some special network topologies. In Ref.^15^, for a special class of intersession networks formed by the cascade of butterfly networks, a construction method of a network coding scheme with better throughput was proposed by using a random linear network coding strategy and combining an evolutionary computation method. In Ref.^16^, the conditions that the three unicast networks with the connecting level vector (*k*_1_, *k*_2_, *k*_3_) needs to meet when using network coding to achieve the unit data transmission rate were analyzed. Except for three unicast networks with a connectivity level vector (2, 2, 4), all types of three unicast networks that meet the constraint condition $\mathop {\min }\limits_{1 \le i \le 3} \{ k_{i} \}$ ≤ 3 were analyzed. For each type, either a network coding data transmission scheme implementing the unit data transmission rate or a counter example that cannot be achieved was given. In Ref.^17^, the degree of freedom of wireless networks with channel interference was studied, and the technology of implementing precoding at the senders and using interference alignment at the receivers to eliminate interference data were proposed. The research idea in Ref.^17^ was drawn upon in Ref.^18^, a special class of three unicast session networks was studied, and a construction method of network coding was proposed. In the three unicast sessions they studied, the maximum flow from each sender to each receiver was one. In this study, a precoding strategy was adopted at the senders, interference alignment technology was used at the receivers, the graph theory method and the algebraic method were used to describe various network topology characteristics, which were summed as the network coupling relationship, and network coding construction in different situations was realized according to the network topology coupling relationship.

Inspired by the research in Ref.^18^, in this study, another special class of three unicast sessions, in which the maximum flow from each sender to each receiver is the same positive integer *k*, and *k* ≥ 2, is investigated. Through analysis and derivation, a feasible network coding construction algorithm is proposed, and it extends the application scope of Ref.^18^. The basic idea is to adopt a multigeneration mixed strategy and select consecutive 2 × *n* + 1 (*n* is a positive integer) generations as a mixed set. In each generation, a precoding strategy is adopted at the senders, and the intermediate nodes implement random linear network coding technology to transmit data. The receivers accept the data of each generation and store them. When the transmission of a mixed set is completed, each receiver combines the data collected by each generation to generate a linear equations system. At each receiver, with the help of the precoding matrices of the senders, interference alignment technology is adopted to eliminate part of the interference information so that the linear equations system can be solved. The proposed algorithm can construct a feasible linear network coding scheme, in which the transmission rate vector of the senders is [(*n* + 1) × *k*/(2 × *n* + 1), *n* × *k*/(2 × *n* + 1), *n* × *k*/(2 × *n* + 1)]. When *n* is sufficiently large, the transmission rate vector asymptotically reaches (*k*/2,* k*/2, *k*/2). The feasibility and correctness of the algorithm are theoretically deduced and mathematically proven. The simulation results verify the conclusion of the theoretical analysis.

## Relevant knowledge and problem description

### Working mechanism of network coding for three unicast sessions

**Figure 1:**
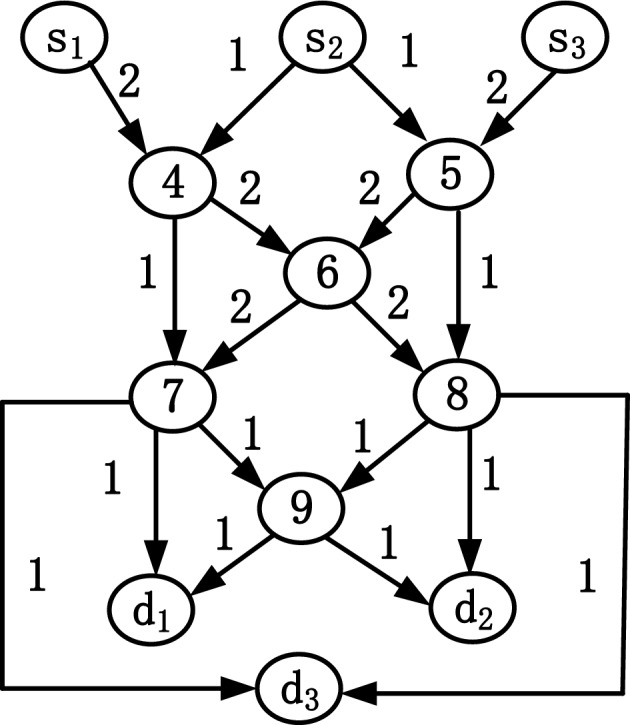
Example of three unicast sessions.

According to the description in Ref.^18^, a network can be represented by a directed acyclic graph *G* = (*V*, *E*), where *V* is the node set and *E* the directed edge set. The network we are considering has three unicast sessions. The *r*th (*r* = 1, 2, 3) unicast session is represented by a tuple *w*_*r*_ = (*s*_*r*_, *d*_*r*_, *X*_*r*_), where *s*_*r*_ and *d*_*r*_ are the sender and the receiver of the *r*th unicast session, respectively. ${\varvec{X}}_{r} = \,\left( {x_{r,1} ,x_{r,2} ,..,x_{{r,h_{r} }} } \right)^{{\text{T}}}$ is a vector of independent random variables, each of which represents a packet that *s*_*r*_ sends to *d*_*r*_ in a generation (also referred to as a time slot), where *x*_*r*,*j*_ ∈ GF (2^*m*^) (*r* = 1, 2, 3 and *j* = 1, 2, …, *h*_*r*_). The network we consider is further restricted. Specifically, the maximum flow from each sender to each receiver is the same positive integer *k*. Figure 1 shows an example of such a network. It is not difficult to verify from the figure that there are three unicast sessions in the network. The maximum flow from each sender to each receiver is 2.

For this network, we intend to adopt a random linear network coding strategy to transmit data. Each node (including the sender) performs a random linear network coding operation on the characters received from its input channels. For each channel *e*_*i*_ ∈ *E*, tail (*e*_*i*_) is the tail node of channel *e*_*i*_, head (*e*_*i*_) is the head node of channel *e*_*i*_, and the characters transmitted on the channel are represented as ${\varvec{y}}_{{{\text{e}}_{i} }}$. Then, according to the linear network coding principle described in Ref.^3^, the character transmitted by channel *e*_*i*_ can be calculated by Formula (1).

1 $${\user2{y}}_{{{\text{e}}_{i} }} = \sum\limits_{{\{ {\text{e}}_{j} |head{\text{(e}}_{j} {)} = {\text{tail}} {\text{(e}}_{i} )\} }} {f_{j,i} {\user2{y}}_{{{\text{e}}_{j} }} }$$

where $\{ {\text{f}}_{j,i} {|}{\text{head}} (e_{j}) = {\text{tail}} (e_{i} )\}$ represents the local coding vector of channel* e*_*i*_. According to the topological characteristics of the directed acyclic graph, through recursive and iterative operations, the character transmitted by each channel can be expressed as the linear combination of the characters sent by each sender, expressed by Formula (2).

2 $${\varvec{y}}_{{{\text{e}}_{i} }} = \sum\limits_{r = 1}^{3} {\Gamma_{e,r} } X_{r}$$

(${\varvec{\varGamma}}_{e,1} ,{\varvec{\varGamma}}_{e,2} ,{\varvec{\varGamma}}_{e,3}$) is the global coding vector of channel *e*_*i*_. For the random linear network coding strategy, each intermediate node should not only transmit the encoded characters to the output channel but also transmit the global coding vector.

Due to the restriction of the maximum flow, each receiver has at least *k* input channels. The receiver *d*_*i*_ (1 ≤ *i* ≤ *3*) collects the global coding vector, carries characters from its input channels and forms a linear equation system according to the corresponding relationship of Formula (2). The linear equation system can be shown by Formula (3).

3 $${\varvec{Y}}_{{\varvec{i}}} = \sum\limits_{j = 1}^{3} {{\varvec{H}}_{j,i} {\varvec{X}}_{{\varvec{j}}} }$$

where *H*_*j*,*i*_ is the transmission gain matrix from *s*_*j*_ to *d*_*i*_, which is only related to the transmission path from *s*_*j*_ to *d*_*i*_. Note that Formula (3) is a representation of the information received by *d*_*i*_ (1 ≤ *i* ≤ 3) in a generation.

The following illustrates the fact that the rank of matrix *H*_*j,i*_ will be equal to the maximum flow from s_*j*_ to *d*_*i*_ with probability close to 1.

#### Theorem 1

In a multiunicast network with *three* unicast sessions, ⑴ the maximum flow from any sender *s*_*j*_ (*j* = 1, 2, 3) to any receiver *d*_*i*_ (*i* = 1, 2, 3) is the same positive integer *k*. ⑵ The selected finite field GF (2^*m*^) has a sufficiently large order. ⑶ The transmission rate of each sender is set to *k* (each sender sends *k* characters to the network in a generation). (4) The random linear network coding strategy is adopted to select the local coding vector of each channel (as shown in Formula (1)) to form a network coding data transmission scheme. Then, under the constructed transmission scheme, the *i*th receiver obtains a linear equation system as shown in Formula (3), and the rank of *H*_*j, i*_ in the linear equation system will reach *k* with a probability close to 1.

#### Proof

After the local coding vectors of each channel are selected by a random linear network coding strategy on the given network, a network coding data transmission scheme is obtained. According to the transmission scheme, the linear equation system shown in Formula (3) is obtained for receiver *d*_*i*_. For a sender *s*_*j*_ (*j* = 1, 2, 3), based on the transmission scheme obtained above, a new network coding data transmission scheme is constructed. In the new scheme, the local coding vector of a channel in the network is set as follows: the local coding vector of the output channel of the sender *s*_*k*_ (*k* ≠ *j*) is set to a zero vector, while the local coding vector of the other channels is the same as that in the original scheme. Then, for the new transmission scheme, the linear equation system obtained by receiver *d*_*i*_ must be shown in Formula (4).

4 $${\varvec{Y}}_{i}{\prime} = {\varvec{H}}_{j,i} {\varvec{X}}_{j}$$

Comparing Formulas (3) and (4), the relevant items of the information sent by *s*_*j*_ in Formula (3) are retained in Formula (4). However, in the new transmission scheme, the local coding vector of the output channel of the other senders is set to a zero vector, so the items associated with the other senders will not appear. On the other hand, the new transmission scheme is equivalent to transmitting data with random linear network coding from *s*_*j*_ to the *receivers*, while the other senders do not transmit data, and *H*_*j*,* i*_ in Formula (4) is the global coding matrix obtained by receiver *d*_*i*_*.* According to the relevant theory of single-sender random linear network coding technology in Ref.^5^, when the selected finite field is sufficiently large, the rank of matrix *H*_*j*,* i*_ will reach *k* with probability close to 1.

Receiver *d*_*i*_ only needs to obtain message *X*_*i*_ sent by sender *s*_*i*_. In receiver *d*_*i*_,*X*_*i*_ can only be calculated by solving the linear equation system in Formula (3), and the linear equation system contains the messages sent by the other senders. In this way, the messages of the other senders are the interference messages of receiver *d*_*i*_. The key to the problem is how to obtain message *X*_*i*_ by solving the linear equation system at receiver *d*_*i*_. In other words, the key to this problem is how to obtain *X*_*i*_ by eliminating the unknown element *X*_*j*_ (*j* ≠ *i*) representing the interference message in the linear equations system.

### Multigeneration mixed strategy

In the process of network coding data transmission, in a generation, after the senders send data, the receivers can receive the coding of the data. There are two ways to decode for the receiver^19^. First, in a generation, each receiver combines the received information to form a linear equations system and immediately solves the linear equations system to recover the sender's data. This method is referred to as the generation-by-generation strategy. Second, if all receivers have a function to store data, then multiple consecutive generations are taken as the decoding unit, in which the receivers store the received information of each generation. When the data transmission in a mixed set is completed, the receivers combine the information received by multiple generations to form a linear equation system and uniformly solve the linear equation system to recover the message of the senders. This method is referred to as the multigeneration mixed strategy, and when multiple consecutive generations are the decoding unit, it is referred to as a mixed set.

### Contributions of this paper

For a special class of three unicast sessions, where the maximum flow from each sender to each receiver is one, the network coding construction algorithm is given in Ref.^18^. Based on their research results, we expand the research scope. Our research object is another special class of three unicast sessions, where the maximum flow from each sender to each receiver is the same positive integer *k*, and *k* ≥ 2. We adopt some of the technology adopted in Ref.^18^, but the main difference is that we use the property of the block matrix and use the Schwarz–Zippel theorem^20^ to prove that the coefficient matrix of the linear equation system is invertible with a probability close to 1.

It is worth noting that the three unicast sessions we are considering cannot be efficiently solved by the method proposed in Ref.^18^. If a virtual sender is added for each sender and a virtual channel with unit capacity is added to connect a virtual sender to its corresponding sender, a new three unicast session is formed, in which the maximum flow from each virtual sender to each receiver is one. The modified three unicast sessions can be solved by using the method in Ref.^18^, but it obviously wastes network transmission resources because the asymptotic data transmission rate vector of the obtained network coding scheme is only (1/2, 1/2, 1/2), whereas the asymptotic data transmission rate vector of the network coding scheme constructed by the proposed method in this paper can reach (*k*/2, *k*/2, *k*/2).

## Network coding construction algorithm

### Analysis and derivation

Because the maximum flow of each sender-receiver pair is the same positive integer *k*, according to the maximum flow minimum cut theorem, the transmission rate of each sender cannot exceed *k*. After selecting the finite field GF (2^*m*^), each sender can send at most *k* characters of GF (2^*m*^) in a generation. However, due to interference in the data transmission process, it is difficult for the data transmission rate to reach *k*. We adopt the multigeneration mixed strategy. First, we select a positive integer *n*. For convenience of writing, remember that *p* = 2 × *n* + 1, *q* = *n* × *k*, and *l* = *p* × *k*. Take *p* consecutive generations as a mixed set. In a mixed set, senders *s*_1_, *s*_2_ and *s*_3_ need to send *q* + *k*, *q* and *q* characters to the network, respectively. The characters to be transmitted from *s*_1_, *s*_2_ and *s*_3_ in the mixed set are formed into three vectors, which are recorded as *X*_1_, *X*_2_ and *X*_3_, respectively, and further written in the following form: ${\varvec{X}}_{i} = (x_{i,1} ,x_{i,2} ,...,x_{{i,h_{i} }} )^{{\text{T}}}$, where *x* ∈ GF(2^*m*^) (*i* = 1, 2, 3, *j* = 1…,*h*_*i*_, *h*_1_ = *q* + *k* and *h*_2_ = *h*_3_ = *q*). At the initial time of each mixed set, each sender *s*_*i*_ (*i* = 1,2,3) implements a precoding strategy, that is, according to the encoding rule of Eq. (5), *s*_*i*_ obtains *l* characters encoded from *X*_*i*_. The encoded *l* characters form a vector that is recorded as $\hat{\user2{X}}_{i} = \left( {\hat{x}_{i,1} ,\hat{x}_{i,2} ,...,\hat{x}_{i,l} } \right)^{{\text{T}}}$. The precoding rule is shown in Formula (5):

5 $$\hat{x}_{i,j} = \mathop \sum \limits_{r = 1}^{{h_{i} }} v_{i,j,r} x_{i,j}$$

where *i* = 1, 2, …, *l*, *j* = 1, 2, …, *h*_*i*_*,* and $v_{i,j,r}$ ∈ GF(2^*m*^).

Formula (5) is rewritten in the form of a matrix product to obtain Formula (6), where *V*_*i*_ is shown in Formula (7).

6 $$\hat{\user2{X}}_{i} = {\varvec{V}}_{i} {\varvec{X}}_{i}$$

7 $${\varvec{V}}_{i} = \left( \begin{gathered} v_{i,1,1} \, v_{{i{,1,2}}} \, ... \, v_{{i{,1,}h_{i} }} \hfill \\ v_{i,2,1} \, v_{{i{,2,2}}} \, ... \, v_{{i{,2,}h_{i} }} \hfill \\ .......................... \hfill \\ v_{i,l,1} \, v_{{{\text{i,}}l{,2}}} \, ... \, v_{{i,l{,}h_{i} }} \hfill \\ \end{gathered} \right)$$

*V*_*i*_ is the precoding matrix of sender *s*_*i*_. Note that *h*_1_ = *q* + *k* and *h*_2_ = *h*_3_ = *q*, so *V*_1_ is a matrix with* l* rows and (*q* + *k*) columns, while *V*_2_ and *V*_3_ are matrices with *l* rows and *q* columns.

In Formula (6), $\hat{\user2{X}}_{i}$ is a vector composed of *l* characters. If these characters are divided into *p* groups evenly, then each group contains *k* characters. Furthermore, if each group is regarded as a vector and recorded as $\hat{\user2{X}}_{i}^{[t]}$(*t* = 1, 2, …,* p*), then Formula (6) can be expressed in the form of Formula (8).

8 $$\hat{\user2{X}}_{i} = {\varvec{V}}_{i} {\varvec{X}}_{i} = \left( {(\hat{\user2{X}}_{i}^{[1]} )^{{\text{T}}} \, (\hat{\user2{X}}_{i}^{[2]} )^{{\text{T}}} \, ... \, (\hat{\user2{X}}_{i}^{[p]} )^{{\text{T}}} } \right)^{{\text{T}}}$$

where $$\hat{\user2{X}}_{i}^{[t]} = \left( {\hat{x}_{i,(t - 1)k + 1} ,\hat{x}_{i,(t - 1)k + 2} ,...,\hat{x}_{i,tk} } \right)^{{\text{T}}}$$, *i* = 1, 2, 3 and *t* = 1, 2, …, *p*.

A mixed set has *p* generations in which each sender needs to transmit *l* encoded characters. Therefore, in each generation, each sender should transmit *k* encoded characters. In the *t*th (1 ≤ *t* ≤ *p*) generation of the mixed set, *s*_*i*_ needs to transmit *k* characters represented by $\hat{\user2{X}}_{i}^{[t]}$; intermediate nodes of the network adopt a random linear network coding strategy for data transmission. The receiver *d*_*i*_ (*i* = 1, 2, 3) receives the global coding vector and the carried characters from *k* input channels and correlates both according to the rules shown in Formula (3). Then, a linear equation system with *k* equations is obtained. If it is written in matrix form, Formula (9) is obtained:

9 $${\varvec{Y}}_{i}^{{{[}t{]}}} = \sum\limits_{j = 1}^{3} {{\varvec{M}}_{{j{,}i}}^{{{[}t{]}}} \hat{\user2{X}}_{1}^{{{[}t{]}}} }$$

where ${\varvec{Y}}_{i}^{[t]} = \left( {\hat{y}_{i,(t - 1)k + 1} ,\hat{y}_{i,(t - 1)k + 2} ,...,\hat{y}_{i,tk} } \right)^{{\text{T}}}$ is a vector composed of *k* characters and ${\varvec{M}}_{j,i}^{[t]}$ replaces *H*_*j*,*i*_ in Formula (3).${\varvec{M}}_{j,i}^{[t]}$ is the transmission gain matrix from *s*_*j*_ to *d*_*i*_ in the *t*th generation. Formula (9) represents the information received by *d*_*i*_ (*i* = 1, 2, 3) in the *t*th generation and contains *k* equations. When the data transmission of a mixed set is completed, receiver *d*_*i*_ can combine these equations obtained in the mixed set to form a linear equation system with *p* × *k* equations, as shown in Formula (10).

10 $$\left\{ {\begin{array}{*{20}c} {\user2{Y}_{i}^{{[1]}} = \user2{M}_{{1,i}}^{{[1]}} \user2{\hat{X}}_{1}^{{[1]}} + \user2{M}_{{2,i}}^{{[1]}} \user2{\hat{X}}_{2}^{{[1]}} \user2{ + M}_{{3,i}}^{{[1]}} \user2{\hat{X}}_{3}^{{[1]}} } \\ {\user2{Y}_{i}^{{[2]}} = \user2{M}_{{1,i}}^{{[2]}} \user2{\hat{X}}_{1}^{{[1]}} + \user2{M}_{{2,i}}^{{[2]}} \user2{\hat{X}}_{2}^{{[2]}} \user2{ + M}_{{3,i}}^{{[2]}} \user2{\hat{X}}_{3}^{{[2]}} } \\ {..........................................................} \\ {\user2{Y}_{i}^{{[p]}} = \user2{M}_{{1,i}}^{{[p]}} \user2{\hat{X}}_{1}^{{[p]}} + \user2{M}_{{2,i}}^{{[2]}} \user2{\hat{X}}_{2}^{{[p]}} \user2{ + M}_{{3,i}}^{{[2]}} \user2{\hat{X}}_{3}^{{[p]}} } \\ \end{array} } \right.$$

Formula (10) is written in the form of a matrix to obtain:

11 $${\varvec{Y}}_{i} = {\varvec{M}}_{1,i} \hat{\user2{X}}_{1} + {\varvec{M}}_{2,i} \hat{\user2{X}}_{2} + {\varvec{M}}_{3,i} \hat{\user2{X}}_{3}$$

where

12 $${\varvec{Y}}_{i} = \left( {\left( {{\varvec{Y}}_{i}^{[1]} } \right)^{{\text{T}}} \,\left( {{\varvec{Y}}_{i}^{[2]} } \right)^{{\text{T}}} \, ... \, \left( {{\varvec{Y}}_{i}^{[p]} } \right)^{{\text{T}}} } \right)^{{\text{T}}}$$

13 $${\varvec{M}}_{j,i} = \left( \begin{gathered} {\varvec{M}}_{j,i}^{[1]} \, {\mathbf{0}} \, \ldots \, {\mathbf{0}} \hfill \\ {\mathbf{0}} \, {\varvec{M}}_{j,i}^{[2]} \, \cdots \, {\mathbf{0}} \hfill \\ \cdots \cdots \cdots \cdots \cdots \cdots \cdots \hfill \\ {\mathbf{0}} \, {\mathbf{0}} \, \cdots \, {\varvec{M}}_{j,i}^{[p]} \hfill \\ \end{gathered} \right)$$

where *i* = 1, 2, 3 and *j* = 1, 2, 3. Replacing *X*_*i*_ in Eq. (11) with the right side of Eq. (6), we obtain

14 $${\varvec{Y}}_{i} = {\varvec{M}}_{1,i} {\varvec{V}}_{{1}} {\varvec{X}}_{{1}} + {\varvec{M}}_{2,i} {\varvec{V}}_{2} {\varvec{X}}_{2} + {\varvec{M}}_{3,i} {\varvec{V}}_{{3}} {\varvec{X}}_{3}$$

In Formula (13), ${\varvec{M}}_{j,i}$ is a block diagonal square matrix of order *l*, and its main diagonal is composed of *p* square matrices of order *k*, which are, respectively expressed as ${\varvec{M}}_{j,i}^{[t]}$(*t* = 1, 2, …, *p*). The remaining bold "0" represents the *k*-order zero matrix.

#### Theorem 2

If the maximum flow from each sender to each receiver is the same positive integer *k*, a random linear network coding strategy is adopted to transmit data in each generation of a mixed set, and the order of the finite field GF (2^*m*^) is sufficiently large, then the matrix ${\varvec{M}}_{j,i}$ in Formula (13) will be reversible with a probability close to 1.

#### Proof

We note that the maximum flow from *s*_*j*_ to *d*_*i*_ is *k*, and *s*_*j*_ transmits *k* encoded characters to the network in each generation. The intermediate node adopts a random linear network coding strategy to determine the local coding vector of its output channel. In the *t*th (*t* = 1,2…, *p*) generation, the transmission gain matrix from *s*_*j*_ to *d*_*i*_ is ${\varvec{M}}_{j,i}^{[t]}$, which is obtained by receiver *d*_*i*_. According to theorem 1, when the order of the selected finite field GF (2^*m*^) is sufficiently large, the rank of ${\varvec{M}}_{j,i}^{[t]}$ will reach* k* with a probability close to 1.${\varvec{M}}_{j,i}^{[t]}$ is a *k*-order square matrix; therefore, the determinant $\user2{|M}_{j,i}^{[t]} |$ will not be equal to zero with a probability close to 1. On the other hand,${\varvec{M}}_{j,i}$ is a block diagonal square matrix. According to the properties of the block diagonal matrix^16^, the determinant of ${\varvec{M}}_{j,i}$ can be expressed as follows:

15 $$\user2{|M}_{j,i} | = \prod\limits_{t = 1}^{p} {\user2{|M}_{j,i}^{[t]} |}$$

Formula (15) means that the determinant of the block diagonal square matrix is equal to the product of the determinant of each block square matrix on the main diagonal, so $\user2{|M}_{j,i} |$ will not be zero with a probability close to 1, that is, ${\varvec{M}}_{j,i}$ will be reversible with a probability close to 1.

According to the definition of three unicast sessions, receiver *d*_*i*_ only needs to obtain the information vector *X*_*i*_ sent by *s*_*i*_, and the linear equation system obtained by *d*_*i*_ is shown in Formula (14). From Formula (14), it can be seen that the linear equations system has *l* = (2 × *n* + 1) × *k* equations but contains messages from three senders, which are represented as unknown elements in the linear equations system. The number of unknown elements reaches (3*n* + 1) × *k*. Obviously, the number of unknown elements is greater than the number of equations. Therefore, some of the unknown elements should be eliminated for the linear equation system shown in Formula (14). In this paper, the interference alignment strategy proposed in Ref.^17^ is adopted to eliminate some of the unknown elements.

After the data transmission of a mixed set is completed, the linear equation system obtained by each sender is as follows:

16 $${\varvec{Y}}_{1} = {\varvec{M}}_{1,1} {\varvec{V}}_{{1}} {\varvec{X}}_{{1}} + {\varvec{M}}_{2,1} {\varvec{V}}_{2} {\varvec{X}}_{2} + {\varvec{M}}_{3,1} {\varvec{V}}_{{3}} {\varvec{X}}_{3}$$

17 $${\varvec{Y}}_{2} = {\varvec{M}}_{1,2} {\varvec{V}}_{{1}} {\varvec{X}}_{{1}} + {\varvec{M}}_{2,2} {\varvec{V}}_{2} {\varvec{X}}_{2} + {\varvec{M}}_{3,2} {\varvec{V}}_{{3}} {\varvec{X}}_{3}$$

18 $${\varvec{Y}}_{3} = {\varvec{M}}_{1,3} {\varvec{V}}_{{1}} {\varvec{X}}_{{1}} + {\varvec{M}}_{2,3} {\varvec{V}}_{2} {\varvec{X}}_{2} + {\varvec{M}}_{3,3} {\varvec{V}}_{{3}} {\varvec{X}}_{3}$$

Formula (16) represents the linear equation system obtained by *d*_1_. *d*_1_ needs to obtain *X*_1_ by solving the linear equations system. To adopt the interference alignment strategy, we force

19 $${\varvec{M}}_{2,1} {\varvec{V}}_{2} = {\varvec{M}}_{3,1} {\varvec{V}}_{{3}}$$

If Formula (19) holds, Formula (16) can be written as Formula (20).

20 $${\varvec{Y}}_{1} = {\varvec{M}}_{1,1} {\varvec{V}}_{{1}} {\varvec{X}}_{{1}} + {\varvec{M}}_{3,1} {\varvec{V}}_{3} \left( {{\varvec{X}}_{2} \user2{ + X}_{3} } \right)$$

Let ${\varvec{X}}_{1}^{\user2{^{\prime}}} = {\varvec{X}}_{2} + {\varvec{X}}_{3}$. Then, Formula (20) can be expressed as Formula (21).

21 $${\varvec{Y}}_{1} = \left( {{\varvec{M}}_{1,1} {\varvec{V}}_{{1}} \user2{ M}_{3,1} {\varvec{V}}_{3} } \right)\left( \begin{gathered} {\varvec{X}}_{1} \hfill \\ {\varvec{X}}_{1}{\prime} \hfill \\ \end{gathered} \right)$$

where $\left( {{\varvec{M}}_{1,1} {\varvec{V}}_{{1}} \user2{ M}_{3,1} {\varvec{V}}_{3} } \right)$ is the coefficient matrix of the linear equation system in Formula (21). For Formula (21), the number of unknown elements is reduced to *l*, which makes the number of unknown elements equal to the number of equations. Furthermore, Formula (21) contains the message variable *X*_1_ required by *d*_1_.

Formula (17) represents the linear equation system obtained by *d*_2_. The interference alignment strategy is used to force

22 $${\varvec{M}}_{3,2} {\varvec{V}}_{3} = {\varvec{M}}_{1,2} {\varvec{V}}_{{1}} {\varvec{A}}$$

where *A* is a matrix of (*q* + *k*) rows and *q* columns. *A* is obtained by removing the rightmost *k* columns from the identity matrix of order (*q* + *k*). Combining Formula (22) and Formula (17), we obtain

23 $${\varvec{Y}}_{2} = {\varvec{M}}_{1,2} {\varvec{V}}_{{1}} \left( {{\varvec{X}}_{{1}} + {\varvec{AX}}_{3} } \right) + {\varvec{M}}_{2,2} {\varvec{V}}_{2} {\varvec{X}}_{2}$$

According to the definition of *A*,

$${\varvec{AX}}_{3} = \left( {x_{3,1} ,x_{3,2} ,...,x_{3,q} ,0,0,...,0} \right)^{{\text{T}}}$$

In Formula (23), let ${\varvec{X}}_{2}^{\user2{^{\prime}}} = {\varvec{X}}_{1} + {\varvec{AX}}_{3} = \left( {x_{1,1} + x_{3,1} ,x_{1,2} + x_{3,2} ,...,x_{1,q} + x_{3,q} ,x_{1,q + 1} ,...,x_{1,q + k} } \right)^{{\text{T}}}$.

Then, Formula (23) can be expressed as Formula (24).

24 $${\varvec{Y}}_{2} = \left( {{\varvec{M}}_{1,2} {\varvec{V}}_{{1}} \user2{ M}_{2,2} {\varvec{V}}_{2} } \right)\left( \begin{gathered} {\varvec{X}}_{2}{\prime} \hfill \\ {\varvec{X}}_{{2}} \hfill \\ \end{gathered} \right)$$

where $\left( {{\varvec{M}}_{1,2} {\varvec{V}}_{{1}} \user2{ M}_{2,2} {\varvec{V}}_{2} } \right)$ is the coefficient matrix of the linear equation system. For Formula (24), the number of unknown elements is reduced to *l*, which makes the number of unknown elements equal to the number of equations. Furthermore, Formula (24) contains the message variable *X*_2_ required by *d*_2_.

Formula (18) represents the linear equation system obtained by *d*_3_. Using interference alignment technology, we force the following:

25 $${\varvec{M}}_{2,3} {\varvec{V}}_{2} = {\varvec{M}}_{1,3} {\varvec{V}}_{{1}} {\varvec{B}}$$

where *B* is a matrix of (*q* + *k*) rows and *q* columns. *B* is obtained by removing the leftmost *k* columns from the identity matrix of order (*q* + *k*). Combining Formula (25) and Formula (18), we obtain:

26 $${\varvec{Y}}_{3} = {\varvec{M}}_{1,3} {\varvec{V}}_{{1}} \left( {{\varvec{X}}_{{1}} + {\varvec{BX}}_{2} } \right){ + }{\varvec{M}}_{3,3} {\varvec{V}}_{3} {\varvec{X}}_{3}$$

The identity matrix of order (*q* + *k*) is represented as following.

$$\left( {\begin{array}{*{20}c} 1 & 0 & 0 & 0 & \cdots & 0 \\ 0 & 1 & 0 & 0 & \cdots & 0 \\ 0 & 0 & 1 & 0 & \cdots & 0 \\ \cdots & \cdots & \cdots & \cdots & \cdots & \cdots \\ 0 & 0 & 0 & 0 & \cdots & 1 \\ \end{array} } \right)$$

According to the definition of matrix *B*, *B* is represented as following.

$$B = \left( {\begin{array}{*{20}c} 0 & 0 & \cdots & 0 & 0 & 0 & \cdots & 0 \\ \cdots & \cdots & \cdots & \cdots & \cdots & \cdots & \cdots & \cdots \\ 0 & 0 & \cdots & 0 & 0 & 0 & \cdots & 0 \\ 0 & 0 & \cdots & 0 & 1 & 0 & \cdots & 0 \\ 0 & 0 & \cdots & 0 & 0 & 1 & \cdots & 0 \\ \cdots & \cdots & \cdots & \cdots & \cdots & \cdots & \cdots & \cdots \\ 0 & 0 & \cdots & 0 & 0 & 0 & \cdots & 1 \\ \end{array} } \right)$$

Then, *BX*_2_ can be represented as following.

$${\varvec{BX}}_{2} = \left( {0, \cdots ,0{,}x_{2,1} ,x_{2,2} ,...,x_{2,q - 1} ,x_{2,q} } \right)^{{\text{T}}}$$

Similarly, let ${\varvec{X}}_{3}^{\user2{^{\prime}}} = {\varvec{X}}_{1} + {\varvec{BX}}_{2} = \left( {x_{1,1} ,x_{1,2} ,...x_{1,k} ,x_{1,\,\,k + 1} + x_{2,1} ,x_{1,k + 2} + x_{2,2} ,...,x_{1,q + k - 1} + x_{2,q - 1} ,x_{1,q + k} + x_{2,q} } \right)$; then, Formula (26) can be expressed as Eq. (27).

27 $${\varvec{Y}}_{3} = \left( {{\varvec{M}}_{1,3} {\varvec{V}}_{{1}} \user2{ M}_{3,3} {\varvec{V}}_{3} } \right)\left( \begin{gathered} {\varvec{X}}_{3}^{\prime } \hfill \\ {\varvec{X}}_{{3}} \hfill \\ \end{gathered} \right)$$

where $\left( {{\varvec{M}}_{1,3} {\varvec{V}}_{{1}} \user2{ M}_{3,3} {\varvec{V}}_{3} } \right)$ is the coefficient matrix. For Formula (27), the number of unknown elements is reduced to *l*. Furthermore, Formula (27) contains the message variable *X*_3_ required by *d*_3_.

The key is now how to determine the elements of *V*_1_, *V*_2_ and *V*_3_ so that Formulas (20), (23) and (26) can be established simultaneously.

According to theorem 2, ${\varvec{M}}_{j,i}$ is reversible with a large probability, and the inverse matrix of ${\varvec{M}}_{j,i}$ is written as ${\varvec{M}}_{j,i}^{ - 1}$.

From Formula (19), we have

28 $${\varvec{V}}_{2} = {\varvec{M}}_{2,1}^{ - 1} {\varvec{M}}_{3,1} {\varvec{V}}_{{3}}$$

From Formula (23), we have

29 $${\varvec{V}}_{3} = {\varvec{M}}_{3,2}^{ - 1} {\varvec{M}}_{1,2} {\varvec{V}}_{{1}} {\varvec{A}}$$

We combine Formula (29) and Formula (28) to obtain:

30 $${\varvec{V}}_{2} = {\varvec{M}}_{2,1}^{ - 1} {\varvec{M}}_{3,1} {\varvec{M}}_{3,2}^{ - 1} {\varvec{M}}_{1,2} {\varvec{V}}_{{1}} {\varvec{A}}$$

From Formula (25), we have

31 $${\varvec{V}}_{2} = {\varvec{M}}_{2,3}^{ - 1} {\varvec{M}}_{1,3} {\varvec{V}}_{1} {\varvec{B}}$$

We combine Formula (30) and Formula (31) to obtain:

32 $${\varvec{M}}_{2,3}^{ - 1} {\varvec{M}}_{1,3} {\varvec{V}}_{1} \user2{B = M}_{2,1}^{ - 1} {\varvec{M}}_{3,1} {\varvec{M}}_{3,2}^{ - 1} {\varvec{M}}_{1,2} {\varvec{V}}_{1} {\varvec{A}}$$

We multiply the matrix ${\varvec{M}}_{1,3}^{ - 1} {\varvec{M}}_{2,3}$ on both sides of Formula (32) to the left and obtain

33 $${\varvec{V}}_{{1}} \user2{B = M}_{1,3}^{ - 1} {\varvec{M}}_{2,3} {\varvec{M}}_{2,1}^{ - 1} {\varvec{M}}_{3,1} {\varvec{M}}_{3,2}^{ - 1} {\varvec{M}}_{1,2} {\varvec{V}}_{{1}} {\varvec{A}}$$

Let

34 $$\user2{T = M}_{1,3}^{ - 1} {\varvec{M}}_{{2,3}} {\varvec{M}}_{2,1}^{ - 1} {\varvec{M}}_{3,1} {\varvec{M}}_{3,2}^{ - 1} {\varvec{M}}_{1,2}$$

Then, Formula (33) becomes the following form:

35 $${\varvec{TV}}_{1} \user2{A = V}_{1} {\varvec{B}}$$

Formula (35) is a constraint on the elements of *V*_1_. Note the definition of matrices *A* and *B*; then, the meaning of Eq. (35) is as follows: the leftmost *q* column of *TV*_1_ corresponds to the rightmost *q* column of *V*_1_.

To meet the constraints on *V*_1_, *V*_1_ is set as follows:

36 $${\varvec{V}}_{1}^{*} = \left( {\user2{W TW T}^{2} \user2{W }... \, {\varvec{T}}^{n} {\varvec{W}}} \right)$$

where *W* is a matrix with *l* rows and *k* columns, of which the elements are randomly selected in the finite field GF (2^*m*^).

*W* is represented as follows:

37 $$\user2{W} = \left( {\begin{array}{*{20}c} {w_{{1,1}} } & {w_{{{\text{1,2}}}} } & \cdots & {w_{{{\text{1,}}k}} } \\ {w_{{2,1}} } & {w_{{{\text{2,2}}}} } & \cdots & {w_{{{\text{2,}}k}} } \\ \cdots & \cdots & \cdots & \cdots \\ {w_{{l,1}} } & {w_{{l{\text{,2}}}} } & \cdots & {w_{{l{\text{,}}k}} } \\ \end{array} } \right)$$

It is not difficult to verify that ${\varvec{V}}_{1}^{*}$ of Formula (36) meets the constraint of Formula (35). When the elements of *V*_1_ are determined, according to Formula (29), we can obtain

38 $${\varvec{V}}_{{3}}^{*} = {\varvec{M}}_{3,2}^{ - 1} {\varvec{M}}_{1,2} \left( {{\varvec{W}} \, \user2{TW T}^{{\varvec{2}}} {\varvec{W}}... \, {\varvec{T}}^{n - 1} {\varvec{W}}} \right)$$

Substituting the right side of Formula (38) into ${\varvec{V}}_{{3}}$ in Eq. (28), we can obtain:

39 $${\varvec{V}}_{2}^{*} = {\varvec{M}}_{2,1}^{ - 1} {\varvec{M}}_{3,1} {\varvec{M}}_{3,2}^{ - 1} {\varvec{M}}_{1,2} \left( {{\varvec{W}} \, \user2{TW T}^{{\varvec{2}}} {\varvec{W}}... \, {\varvec{T}}^{n - 1} {\varvec{W}}} \right)$$

According to the above derivation, as long as the precoding matrix of each sender is selected according to Formulas (36), (38) and (39), the part of unknown elements in the linear equations system shown in Formulas (16), (17) and (18) can be eliminated and then changed into the form of Formulas (21), (24) and (27), respectively, so that the number of unknown elements in the above linear equations system is exactly equal to the number of equations.

Next, it is proven that the coefficient matrix of the linear equation system shown in Formulas (21), (24) and (27) is a full-rank matrix, which is proven by taking Formula (21) as an example.

#### Theorem 3

When the selected finite field is sufficiently large, each element of the matrix *W* in Eq. (37) is randomly selected, and the precoded matrix is selected according to Formulas (36), (38), and (39), then the coefficient matrix of the linear equations system obtained by *d*_1_ as shown in Formula (21), the coefficient matrix of the linear equations system obtained by *d*_2_ as shown in Formula (24), and the coefficient matrix of the linear equation system obtained by *d*_3_ as shown in Formula (27) will be the full-rank matrix with high probability.

#### Proof

It is only proven that the coefficient matrix of Formula (21) is a full-rank matrix with a high probability. For receiver *d*_1_, the coefficient matrix of the linear equation system as shown in Formula (21) is $\left( {{\varvec{M}}_{{1,1}} {\varvec{V}}_{{1}}^{*} \user2{ M}_{{3,1}} {\varvec{V}}_{{3}}^{*} } \right){ = }{\varvec{M}}_{{1,1}} \left( {{\varvec{V}}_{{1}}^{*} \, {\varvec{M}}_{{1,1}}^{{ - {1}}} {\varvec{M}}_{{1,3}} {\varvec{V}}_{{3}}^{*} } \right)$. According to theorem 1, *M*_1,1_ is a full-rank matrix; thus, it is only necessary to prove that ${\varvec{S}}{ = }\left( {{\varvec{V}}_{1}^{*} \, {\varvec{M}}_{1,1}^{ - 1} {\varvec{M}}_{1,3} {\varvec{V}}_{3}^{*} } \right)$ is a full-rank matrix with a high probability. Furthermore, it is only necessary to prove that the determinant of *S* is not zero with a high probability. For *S*, replace ${\varvec{V}}_{1}^{*}$ and ${\varvec{V}}_{3}^{*}$ with the right side of Eq. (36) and the right side of Eq. (38), respectively, and further let ${\varvec{D}} = {\varvec{M}}_{1,1}^{ - 1} {\varvec{M}}_{1,3} {\varvec{M}}_{3,2}^{ - 1} {\varvec{M}}_{1,2}$; then,

40 $${\varvec{S}}{ = }\left( {{\varvec{W}} \, {\varvec{TW}}...{\varvec{T}}^{n} {\varvec{W}} \, {\varvec{DW}} \, \user2{DTW }... \, {\varvec{DT}}^{{n{ - 1}}} {\varvec{W}}} \right)$$

In Formula (40), after determining the elements in matrices *T* and *D*, these elements are regarded as constants. Since the elements in *W* are randomly selected, they are regarded as unknown elements. Let $W = (w_{i,j} )_{l \times k}$, which has a total of *l* × *k* elements. From the composition of each block matrix of *S* in Formula (40), it can be seen that the elements in *S* are either *w* (such as the elements in the first *k* column) or a linear combination of elements in a column of *W* (such as *TW* forming the *k* column of *S*, and each element in the *k* column is a linear combination of elements in a column of *W*). Therefore, an element in *S* is a multivariate primary polynomial with respect to the unknown element *w*_*i*,* j*_. Next, we examine the determinant of *S*. According to the properties of the expansion of the determinant and note that *S* is a matrix of *l* order. Then, $|{\varvec{S}}|$ is the algebraic sum of the expansion items, where each item is the product of *l* elements in the matrix *S* that are not in the same row and the same column. Thus, $|{\varvec{S}}|$ is a multivariate polynomial with respect to the unknown elements *w*_*i*,*j*_ (*i* = 1, 2, …, *l* and *j* = 1, 2, …, *k*). Since the highest degree of each element in the matrix *S* is one with respect to *w*_i,j_, the highest degree of the multivariate polynomial of $|{\varvec{S}}|$ is *l* with respect to *w*_*i, j*_. We write this polynomial as *f* (*w*_1,1_, *w*_1,2_, …, *w*_1, *k*_, …, *w*_*l, k*_), and its degree does not exceed *l*. However, *w*_*i*,* j*_ are randomly taken in GF (2^*m*^). Therefore, according to the Schwarz–Zippel theorem^20^, the probability of polynomial* f* not being zero is greater than $1 - \frac{l}{{2^{m} }}$. Therefore, when the order of the finite field is relatively large, the probability of the determinant not being 0 is quite large, so the coefficient matrix of Formula (21) will be a full-rank matrix with a large probability. In the same way, it can be proven that the coefficient matrix of Formula (24) and Formula (27) is a full-rank matrix with a high probability.

Since the probability of the coefficient matrix of the linear equation system is high, it can be ensured that the coefficient matrix is a full rank matrix as long as the elements of *W* are randomly selected several times.

### Algorithm description

**Algorithm 1**: A coding construction algorithm for a special class of three unicast sessions.

- **Input**: Topology information of a given network.
  - **Step 1**: The algorithm is initialized. A finite field GF (2^*m*^) and a positive integer *n* are selected, and *p* = 2 × *n* + 1, *q* = *n* × *k,* and *l* = *p* × *k*. Take *p* consecutive generations as a mixed set. In the mixed set, *s*_1_, *s*_2_, and *s*_3_ should transmit *q* + *k*, *q*, and *q* characters to the network, respectively. Each sender precodes the characters to be sent in a mixed set to form *1* coding character.
  - **Step 2**: For each generation of a mixed set, each sender sends *k* precoded characters to the network. A random linear network coding strategy is used to determine the local coding vector of each channel. Each receiver accepts the information from its input channels and stores it.
  - **Step 3**: When data transmission is completed in a mixed set, each receiver collects the information obtained at each generation in the mixed set to form a linear equation system, as shown in Formula (10).
  - **Step 4**: The matrix *T* is calculated according to Formula (34).
  - **Step 5**: The elements of matrix *W* in Formula (37) are randomly selected.
  - **Step 6**: The precoding matrix of each sender is calculated according to Formulas (36), (38) and (39).
  - **Step 7:** The coefficient matrices of Formulas (21), (24) and (27) are calculated, and a determination of whether they are full rank matrices is made. If they are full rank matrices, the algorithm ends; otherwise, go to step 5.
- **Output**: Network coding data transmission scheme.

The above algorithm can obtain a network coding data transmission scheme for a special class of three unicast sessions. In this scheme, the transmission rate vector of the senders is ($\frac{l + k}{p}$,$\frac{l}{p}$,$\frac{l}{p}$) = [$\frac{{\left( {n + 1} \right) \times k}}{2 \times n + 1}$,$\frac{n \times k}{{2 \times n + 1}}$,$\frac{n \times k}{{2 \times n + 1}}$].

## Simulation test

A simulation test is carried out on the network shown in Fig. 1. It can be seen from the figure that *k* = 2. Let *n* = 1, and take* p* = 2*n* + 1 = 3 generations as a mixed set. Then, *q* = *n* × *k* = 2, and *l* = *p* × *k* = *6*. The Galois field GF (2^3^), whose irreducible polynomial is *x*^3^ + *x* + 1, is selected and written in binary form as (1011)_2_. The number of characters sent by the senders in a mixed set forms a vector, which is (*q* + *k*, *q*, *q*) = (4, 2, 2). The intermediate nodes use a random linear network coding strategy to transmit data. The following is the transmission gain matrix obtained from a mixed set.

The three block matrices on the main diagonal of *M*_1,1_ are:

$$\left( {\begin{array}{*{20}c} {010} & {011} \\ {100} & {110} \\ \end{array} } \right),\,\left( {\begin{array}{*{20}c} {100} & {100} \\ {011} & {111} \\ \end{array} } \right),\,\left( {\begin{array}{*{20}c} {100} & {100} \\ {011} & {111} \\ \end{array} } \right)$$

The three block matrices on the main diagonal of *M*_1,2_ are:

$$\left( {\begin{array}{*{20}c} {000} & {000} \\ {110} & {111} \\ \end{array} } \right),\,\left( {\begin{array}{*{20}c} {010} & {010} \\ {100} & {001} \\ \end{array} } \right),\,\left( {\begin{array}{*{20}c} {010} & {010} \\ {100} & {001} \\ \end{array} } \right)$$

The three block matrices on the main diagonal of *M*_1,3_ are:

$$\left( {\begin{array}{*{20}c} {101} & {100} \\ {110} & {111} \\ \end{array} } \right),\,\left( {\begin{array}{*{20}c} {110} & {111} \\ {110} & {110} \\ \end{array} } \right),\,\left( {\begin{array}{*{20}c} {110} & {111} \\ {110} & {110} \\ \end{array} } \right)$$

The three block matrices on the main diagonal of *M*_2,1_ are:

$$\left( {\begin{array}{*{20}c} {111} & {110} \\ {101} & {101} \\ \end{array} } \right),\left( {\begin{array}{*{20}c} {100} & {100} \\ {011} & {000} \\ \end{array} } \right),\,\left( {\begin{array}{*{20}c} {100} & {100} \\ {011} & {000} \\ \end{array} } \right)$$

The three block matrices on the main diagonal of *M*_2,2_ are:

$$\left( {\begin{array}{*{20}c} {011} & {010} \\ {110} & {111} \\ \end{array} } \right),\,\left( {\begin{array}{*{20}c} {001} & {001} \\ {100} & {101} \\ \end{array} } \right),\,\left( {\begin{array}{*{20}c} {001} & {001} \\ {100} & {101} \\ \end{array} } \right)$$

The three block matrices on the main diagonal of *M*_2,3_ are:

$$\left( {\begin{array}{*{20}c} {000} & {011} \\ {010} & {001} \\ \end{array} } \right),\,\left( {\begin{array}{*{20}c} {010} & {010} \\ {011} & {100} \\ \end{array} } \right),\,\left( {\begin{array}{*{20}c} {010} & {010} \\ {011} & {100} \\ \end{array} } \right)$$

The three block matrices on the main diagonal of *M*_3,1_ are:

$$\left( {\begin{array}{*{20}c} {010} & {011} \\ {010} & {011} \\ \end{array} } \right),\left( {\begin{array}{*{20}c} {010} & {001} \\ {001} & {110} \\ \end{array} } \right),\,\left( {\begin{array}{*{20}c} {010} & {001} \\ {001} & {110} \\ \end{array} } \right)$$

The three block matrices on the main diagonal of *M*_3,2_ are:

$$\left( {\begin{array}{*{20}c} {010} & {011} \\ {010} & {011} \\ \end{array} } \right),\,\left( {\begin{array}{*{20}c} {010} & {001} \\ {001} & {110} \\ \end{array} } \right),\,\left( {\begin{array}{*{20}c} {010} & {001} \\ {001} & {110} \\ \end{array} } \right)$$

The three block matrices on the main diagonal of *M*_3,3_ are:

$$\left( {\begin{array}{*{20}c} {010} & {011} \\ {010} & {011} \\ \end{array} } \right),\,\left( {\begin{array}{*{20}c} {010} & {001} \\ {001} & {110} \\ \end{array} } \right),\,\left( {\begin{array}{*{20}c} {010} & {001} \\ {001} & {110} \\ \end{array} } \right)$$

The matrix *T* is calculated according to Formula (34), and the three block matrices on the main diagonal of *T* are:

$$\left( {\begin{array}{*{20}c} {010} & {011} \\ {010} & {011} \\ \end{array} } \right),\,\left( {\begin{array}{*{20}c} {010} & {001} \\ {001} & {110} \\ \end{array} } \right),\,\left( {\begin{array}{*{20}c} {010} & {001} \\ {001} & {110} \\ \end{array} } \right)$$

The elements in *W* are randomly selected. *W* is a matrix of 6 rows and 2 columns. The elements of *W* are as follows:

$$\user2{W} = \left( {\begin{array}{*{20}c} {001} & {100} & {001} & {011} & {100} & {111} \\ {110} & {010} & {101} & {100} & {011} & {001} \\ \end{array} } \right)^{{\text{T}}}$$

${\varvec{V}}_{1}^{*}$, ${\varvec{V}}_{2}^{*}$, and ${\varvec{V}}_{3}^{*}$ are calculated according to Formulas (35), (38) and (39), respectively, to obtain

$$\user2{V}_{1}^{*} = \left( {\begin{array}{*{20}c} {110} & {001} & {011} & {010} & {101} & {100} \\ {011} & {000} & {101} & {011} & {011} & {100} \\ {011} & {000} & {011} & {110} & {101} & {100} \\ {110} & {000} & {101} & {111} & {011} & {101} \\ \end{array} } \right)^{{\text{T}}}$$

$$\user2{V}_{{\text{2}}}^{{\text{*}}} = \left( {\begin{array}{*{20}c} {010} & {101} & {011} & {100} & {011} & {010} \\ {100} & {001} & {001} & {001} & {010} & {000} \\ \end{array} } \right)^{{\text{T}}}$$

$$\user2{V}_{3}^{*} = \left( {\begin{array}{*{20}c} {011} & {000} & {101} & {000} & {000} & {100} \\ {110} & {000} & {101} & {001} & {010} & {111} \\ \end{array} } \right)^{{\text{T}}}$$

The coefficient matrix of the linear equations system obtained by *d*_1_ is:

41 $$\begin{gathered} \left( {\user2{M}_{{1,1}} \user2{V}_{1}^{*} {\text{ }}\user2{M}_{{2,1}} \user2{V}_{2}^{*} {\text{ }}\user2{M}_{{3,1}} \user2{V}_{3}^{*} } \right) = \hfill \\ \left( {\begin{array}{*{20}c} {100} & {110} & {110} & {111} & {110} & {111} & {110} & {111} \\ {011} & {111} & {111} & {101} & {110} & {111} & {110} & {111} \\ {100} & {101} & {010} & {011} & {001} & {000} & {001} & {000} \\ {000} & {110} & {001} & {111} & {101} & {011} & {101} & {011} \\ {100} & {001} & {100} & {100} & {100} & {011} & {100} & {011} \\ {101} & {100} & {101} & {000} & {101} & {110} & {101} & {110} \\ \end{array} } \right) \hfill \\ \end{gathered}$$

From the coefficient matrix shown in Formula (41), it can be seen that the fifth column is the same as the seventh column, and the sixth column is the same as the eighth column. Note that the unknown elements corresponding to Columns 5 and 6 are *x*_2,1_ and *x*_2,2_, respectively, and the unknown elements corresponding to Columns 7 and 8 are *x*_3,1_ and *x*_3,2_, respectively. Let $x_{1,1}^{\user2{^{\prime}}} = x_{2,1} + x_{3,1}$ and $x_{1,2}^{\user2{^{\prime}}} = x_{2,2} + x_{3,2}$; then, the linear equation system has six equations and six unknown elements. After taking the first, second, third, fourth, seventh and eighth columns of the matrix in Formula (41), the coefficient matrix of the linear equation system after elimination is obtained as follows:

42 $$\begin{gathered} \left( {\user2{M}_{{1,1}} \user2{V}_{1}^{*} \,\user2{M}_{{3,1}} \user2{V}_{3}^{*} } \right) = \hfill \\ \left( {\begin{array}{*{20}c} {100} & {110} & {110} & {111} & {110} & {111} \\ {011} & {111} & {111} & {101} & {110} & {111} \\ {100} & {101} & {010} & {011} & {001} & {000} \\ {000} & {110} & {001} & {111} & {101} & {011} \\ {100} & {001} & {100} & {100} & {100} & {011} \\ {101} & {100} & {101} & {000} & {101} & {110} \\ \end{array} } \right) \hfill \\ \end{gathered}$$

It is not difficult to verify that the coefficient matrix in Formula (42) is a full-rank matrix, so the linear equations system can be solved. The solution results are $x_{1,1}$, $x_{1,2}$, $x_{1,3}$, $x_{1,4}$, $x_{1,1}^{\user2{^{\prime}}}$, and $x_{1,2}^{\user2{^{\prime}}}$, of which the first four messages are all from sender *s*1, which is exactly what receiver *d*_1_ needs, and the last two are interference messages.

The coefficient matrix of the linear equations system obtained at receiver *d*_2_ is

43 $$\begin{gathered} \left( {\user2{M}_{{1,2}} \user2{V}_{1}^{*} {\text{ }}\user2{M}_{{2,2}} \user2{V}_{2}^{*} {\text{ }}\user2{M}_{{3,2}} \user2{V}_{3}^{*} } \right) = \hfill \\ \left( {\begin{array}{*{20}c} {000} & {000} & {000} & {000} & {011} & {110} & {000} & {000} \\ {101} & {001} & {001} & {010} & {001} & {010} & {101} & {001} \\ {010} & {111} & {001} & {100} & {111} & {000} & {010} & {111} \\ {101} & {001} & {001} & {101} & {101} & {001} & {101} & {001} \\ {010} & {101} & {010} & {010} & {001} & {010} & {010} & {101} \\ {110} & {011} & {110} & {101} & {110} & {011} & {110} & {011} \\ \end{array} } \right) \hfill \\ \end{gathered}$$

From the coefficient matrix shown in Formula (43), it can be seen that the first and second columns are the same as the seventh and eighth columns, respectively, so two unknown elements can be eliminated, and the first to sixth columns can be retained to form a new linear equation system of six equations and six unknown elements. The unknown elements corresponding to the fifth and sixth columns are sent from sender *s*_2_, so the characters sent by sender s_2_ can be obtained after solving the linear equations system.

The coefficient matrix of the linear equations system obtained at receiver *d*_3_ is

44 $$\begin{gathered} \left( {\user2{M}_{{1,3}} \user2{V}_{1}^{*} \user2{ M}_{{2,3}} \user2{V}_{2}^{*} \user2{ M}_{{3,3}} \user2{V}_{3}^{*} } \right) = \hfill \\ \left( {\begin{array}{*{20}c} {111} & {100} & {100} & {011} & {100} & {011} & {001} & {010} \\ {101} & {001} & {001} & {010} & {001} & {010} & {110} & {111} \\ {100} & {001} & {101} & {000} & {101} & {000} & {101} & {000} \\ {110} & {010} & {011} & {111} & {011} & {111} & {000} & {001} \\ {010} & {000} & {010} & {100} & {010} & {100} & {010} & {100} \\ {110} & {100} & {110} & {110} & {110} & {110} & {100} & {111} \\ \end{array} } \right) \hfill \\ \end{gathered}$$

From the coefficient matrix shown in Formula (44), it can be seen that the fifth and sixth columns are the same as the third and fourth columns, respectively, so two unknown elements can be eliminated, and the first to fourth columns and the seventh to eighth columns can be retained to form a new linear equation system of six equations and six unknown elements. The unknown elements corresponding to the last two columns are sent from sender s3, so the characters sent by sender s3 can be obtained after solving the linear equations system.

In summary, a network coding data transmission scheme, in which the transmission rate vector of the senders is (4/3, 2/3, 2/3), is obtained. Due to space constraints, the value of *n* is smaller. When the value of *n* is sufficiently large, the transmission rate vector of the sender is close to (*k*/2, *k*/2, *k*/2).

## Conclusion

Inspired by the research idea in Ref.^18^, a feasible construction algorithm of a network coding data transmission scheme is proposed for a special class of three unicast sessions. The three unicast sessions we consider have the following characteristics: the maximum flow from each sender to each receiver is the same positive integer *k*. Our study extends the scope of application in Ref.^18^. A multigeneration mixed strategy is adopted, and there are (2 × *n* + 1) generations in a mixed set. At the senders, all characters that need to be transmitted are precoded in a mixed set and mapped to (2 × *n* + 1) × *k* characters. In each generation of the mixed set, each sender transmits precoded *k* characters to the network. In the intermediate nodes, a random linear network coding strategy is adopted to transmit data. The receivers receive and store the data transmitted by each generation. When the data transmission of a mixed set is completed, the receivers collect the data of each generation and form a linear equation system. The precoding of the senders and interference alignment technology are adopted to eliminate interference information so that the linear equation system can be solved. Therefore, the message required by the receiver can be recovered. In this paper, the feasibility and correctness of the algorithm are strictly deduced and proven, and a simulation example is given. The simulation results show the effectiveness of the algorithm.

## Data Availability

All data generated or analysed during this study are included in this published article.

## References

[1] Ahlswede R, Cai N, Li SY, Yeung RW (2000). Network information flow. IEEE Trans. Inform. Theory.

[2] Li SY, Yeung RW, Cai N (2003). Linear network coding. IEEE Trans. Inform. Theory.

[3] Koetter R, Medard M (2003). An algebraic approach to network coding. IEEE/ACM Trans. Netw..

[4] Jaggi S, Sanders P, Chou PA (2005). Polynomial time algorithms for multicast network code construction. IEEE Trans. Inform. Theory.

[5] Ho T, Medard M, Koetter R (2006). A random linear network coding approach to multicast. IEEE Trans. Inform. Theory.

[6] Matsuda T, Noguchi T, Takine T (2011). Survey of network coding and its application. IEICE Trans. Commun..

[7] Aktas T, Yilmaz AO, Aktas E (2013). Practical methods for wireless network coding with multiple unicast transmissions. IEEE Trans. Commun..

[8] Yeung RW, Li SYR, Cai N (2005). Network Coding theory part II: Multiple source. Commun. Inform. Theory.

[9] Abudaqa A, Mahmoud A (2020). Abu-Amaea M Super generation network coding for peer-to-peer content distribution networks. IEEE Access.

[10] Wu C.H., Lo S.T. Network-coded storages for cloud computing. International Conference on Applied System Innovation. IEEE, (2017).

[11] Akilandeswart G., Martin M.L.J. Next Generation Network Coding Technique for IoT. 2020,11th International Conference on Computing, Communication and Networking Technologies (ICCCNT), Kharagpur, India, 2020, pp. 1–6, doi: 10.1109/ICCCNT49239.2020.9225314 (2020).

[12] Zhu F (2021). Practical network coding technologies and softwarization in wireless networks. IEEE Internet Things J..

[13] Dougherty R, Zeger K (2006). Nonreversibility and equivalent constructions of multiple-unicast networks. IEEE Trans. Inform. Theory.

[14] Kamath S, Anantharam V, Tse D (2018). The two-unicast problem. IEEE Trans. Inform. Theory.

[15] Kim, M., Medard, M., Traskov, D. An evolutionary approach to inter-session network coding. In: Proc IEEE Infocom Rio De Janro Brazil April. 450–458 (2009).

[16] Huang S, Ramamoorthy A (2014). On the multiple-unicast capacity of 3-source, 3-terminal directed acyclic networks. IEEE/ACM Trans. Netw..

[17] Cadanbe VR, Jafar SA (2008). Interference alignment and degrees of freedom of the k-user interference channel. IEEE Trans. Inform. Theory.

[18] Meng C, Das AK, Ramakrishnan A (2015). Precoding-based network alignment for three unicast sessions. IEEE Trans. Inform. Theory.

[19] Halloush, M., Radha, H. Network coding with multi-generation mixing: analysis and applications for video communication. IEEE International Conference on Communications (2008).

[20] Motwabi P, Raghavan P (1995). Randomized Algorithms.

